# Ocular Tissue for Research in Australia: Strategies for Potential Research Utility of Surplus and Transplant-Ineligible Deceased Donations

**DOI:** 10.1167/tvst.9.5.4

**Published:** 2020-04-13

**Authors:** Heather Machin, Karl Brown, Gerard Sutton, Paul Baird

**Affiliations:** 1 Lions Eye Donation Service, Centre for Eye Research Australia, Royal Victorian Eye and Ear Hospital, Victoria, Australia; 2 Department of Surgery, Ophthalmology, University of Melbourne, Victoria, Australia; 3 New South Wales Tissue Bank, New South Wales, Australia; 4 Save Sight Institute, Discipline of Ophthalmology, Sydney Medical School, University of Sydney, Sydney, New South Wales, Australia; 5 University of Technology Sydney, Graduate School of Health, New South Wales, Australia

**Keywords:** ocular tissue for research, eye bank, biobank, vision science, human tissue, future therapies

## Abstract

A 2016 Price Waterhouse Cooper Report, commissioned by the Australian Commonwealth Government's Organ and Tissue Authority, indicated that Australia had been meeting its human ocular tissue for transplant needs. It further suggested that Australia should consider exportation as a management strategy for excess tissue. Although we do not seek to discuss how the Price Waterhouse Cooper Report determined that need was being met, nor the potential value of exportation in this article, we propose that Ocular Tissue for Research (OTR), and particularly identification of donors for research, and timely access to fresh domestic tissue, be considered as an alternate or simultaneous surplus management strategy. A robust OTR system could provide long-term domestic support and investment into research and development of therapies in Australia. Such a system would also provide a meaningful donation option for those otherwise unable to donate for transplant. This article attempts to document, for the first time to our knowledge, the current recovery and distribution processes of deceased OTR in Australia. It maps the process steps, identifies the stakeholders and needs, discusses the limitations and barriers, and proposes key policy and practice reform strategies that may assist in improving access to OTR.

**Translational Relevance:**

To improve and increase access to human ocular tissue for research, and in turn, advance vision science and clinical application.

## Introduction

The advancement of vision science and the development of most new treatments, techniques, and diagnostics is dependent on access to sufficient quantities of well-characterized high-quality human ocular tissues. Although the 2016 National Research Infrastructure Roadmap^1^ includes calls for a national framework regarding access to biological samples, to date, there are no specific Ocular Tissue for Research (OTR) national strategies in place to prepare Australia, as it explores new technologies (e.g., tissue engineering and stem cells), as well as provide ocular tissue for conventional research needs (e.g., corneal diseases or retinal material for diabetic eye disease, glaucoma, and age-related macular degeneration).

Although some tissue samples can be obtained from a living human, primarily identified and managed by clinician-scientists and living donation programs, the majority of vision science researchers are reliant on deceased donation from consented voluntary and altruistic donors. This, in turn, has made researchers reliant on the deceased organ and tissue donation sector for donor identification, consent, recovery, processing, and allocation.

As the Australian organ and tissue donation sector has been historically focused, funded, and designed for the purposes of transplant, OTR services were added by way of an ad hoc by-product service, rather than as a direct and unique area of planned service. However, more recent developments through the emergence of biobanks and the 2016 National Research Infrastructure Roadmap are changing this paradigm.

This article highlights the need for a coordinated approach to deceased OTR and prompt collaborative discussions with key stakeholders, to allow planning for OTR use going forward.

## The Current Process

### The Researcher

In Australia, OTR is typically provided to researchers by their closest Australian Eye Bank (AUEB) (Adelaide, Brisbane, Melbourne, Perth, and Sydney) or at times, the Biobank (The Australian Ocular Biobank, Sydney). The researchers must provide evidence of human research ethics approval alongside information about their project, team members, and the type of tissue they require. Depending on the research, they may ask for a certain quantity, tissue type (e.g., the retina or the cornea), and storage preference (fresh or preserved or specific storage condition, for example, liquid nitrogen, –80°C, for nuclear material or subsequent derivation of cell lines from the tissue, and others). At times, they may also request diseased or nondiseased (control) tissue, or tissue from a donor with certain characteristics (age, smoking status, and so on). The researcher is then notified by the AUEB/Biobank if donated OTR matching their request is available.

Due to uneven supply and demand, researchers may go through periods without any OTR, and then be provided with several in quick succession. Researchers are required to plan their experiments around the availability accordingly, often having little time to collect and utilize the tissue before it expires or before another researcher lays claim to the tissue. Although a small minority of researchers do budget for tissue service costs, with preplanned recovery (removal) processes in place with their local AUEB or Biobank, most do not, and rely instead on tissue provided on a “no-cost” basis by the AUEB. This may reflect the research process, for example, additional experiments, changes in protocols, or emergence of new techniques, meaning OTR needs may change after the time of initial research grant application or reflect emerging areas of new research.

### The Organ and Tissue Sector

The process of determining deceased donors for research varies among the Australian States and Territories, although it generally commences with the donor coordinators located in the hospital setting.^2^ They ascertain willingness of an individual to donate for transplant and/or research and training. Willing donors (via their next-of-kin) indicate their donation wish. The donor coordinators notify the AUEB when potential donors are identified.

**Figure 1.**
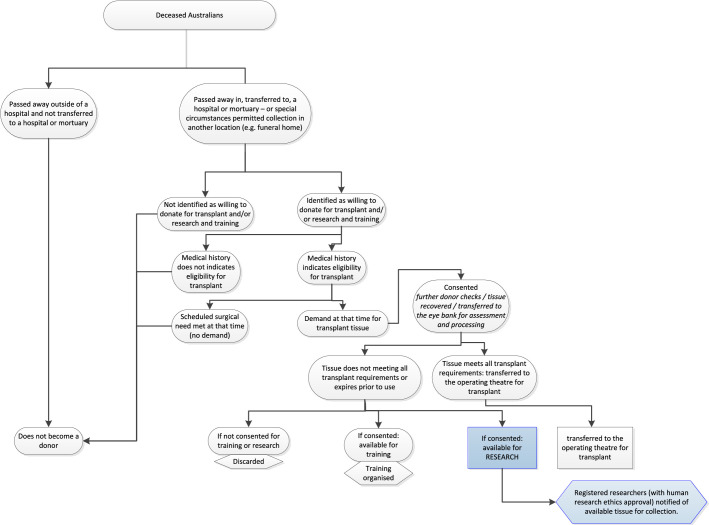
Current OTR pathway in Australia.

As the donation system in Australia is historically designed to support transplantation, only donors who are potential eligible donors for transplantation tend to progress in the system. The donors are then triaged through a donor eligibility criteria pathway before consent and recovery is performed. The eligibility and donation pathway is outlined in Figure 1, demonstrating the various steps necessary for triage toward donation. Those eligible for transplantation are consented (via their next-of-kin) prior to recovery of their donation. This includes examination of medical, surgical, and social donor history. Blood samples analyzed and ocular tissue integrity and suitability are examined closely. Ocular tissue that continues to meet transplant eligibility criteria becomes transplant ocular tissue. If the donor is eligible, and there are scheduled recipients awaiting a transplant, the AUEB recovers the donation. They transfer the donation to the AUEB for processing, and then allocate to a waiting recipient.

**Figure 2.**
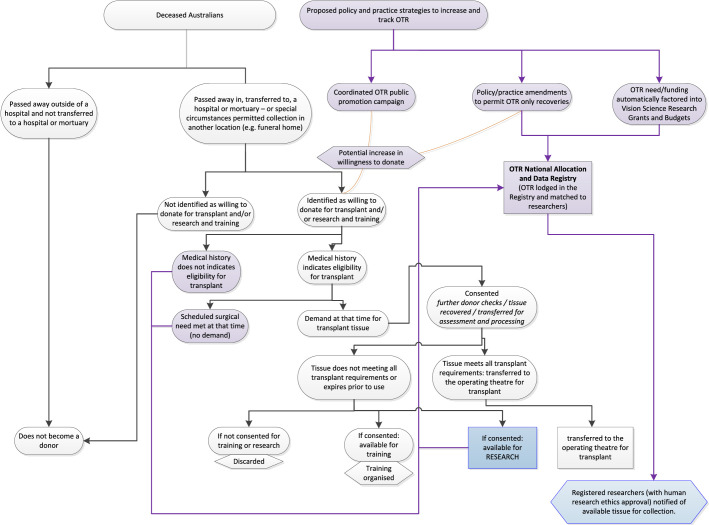
Proposed reform pathway to improve OTR access in Australia. Purple/filled boxes provide proposed strategy.

Recovered ocular tissue that during the examination process is deemed unsuitable for donation, does not progress as transplant tissue. Instead, it becomes potential tissue for training and/or research, however, this can only occur if the donor also consented for research and/or training. Those not consented are discarded.

AUEB's vary in terms of what they recover from a donor, with some collecting just the corneoscleral rim for corneal transplant, or occasionally the whole globe for scleral need. Others routinely collect the whole globe. Although the cornea and sclera are needed for transplantation, the other parts of the whole globe are discarded, unless there are known researchers with Human Research Ethics Committee approval needing the donation and the donor has consented for research use.

Alternatively, if the AUEB does not need tissue at that time, or if the donor is ineligible for transplant, the donor is not moved ahead in the pathway presented in Figure 1. This means that potential OTR is not collected, even if the donor may have considered donation for research only. Furthermore, this indicates a potential pool of OTR donors who could otherwise donate for research, if funding allowed for their recovery by the AUEB.

Historically in Australia, ocular tissue was provided to transplant recipients without charge, with cost recovery services philanthropically supported.^3^ Although philanthropic and benevolent support (such as Lions Clubs) remain, each AUEB is reimbursed for the provision of a transplantable graft (e.g., cornea) by Medicare in Australia or a recipient's health insurance company, with costs outlined on the Australian Prosthesis List. In this model, tissue for transplantation is funded, whereas nontransplant tissue (e.g., OTR) is not funded, resulting in an individual AUEB, or rarely a researcher, incurring the cost.

Each AUEB has established cost recovery systems independently. Some are able to provide the ocular tissue to researchers without a fee, whereas others require a cost recovery fee up to AU$500. Fee structures also take into consideration any preparation (e.g., dissection or provided whole) or preservation cost-recovery needs, to meet the preservation requirements of the researcher.

## Research Needs

To date, there is no centralized registry in Australia regarding OTR utility and need. Although individual AUEBs do record tissue recovered for transplant, that later becomes OTR, their data do not merge with other AUEBs/Biobanks, possible imports, and other nonbank living procurement services, nor do Australian authorities collect data on any human material required or used for research. Therefore despite an Australian national research infrastructure scoping review anticipated to commence in 2020^1^ to review and plan for access and allocation (e.g., via Biobanks), today there is no clear picture of OTR current or future need within Australia.

As an indicator of potential need, research conducted by Stamer et al.,^4^ based in the United States, identified in a voluntary survey of the Association for Researchers in Vision and Ophthalmology's (ARVO) domestic and international members, that vision science researchers had a strong interest in using more tissue if it were easier to obtain. Although no similar study has been performed in Australia, we suspect the same may also be true of Australian researchers, with research size and design adapted (or reduced) to meet OTR availability. As a guide, Stamer et al.^4^ also outlined that their respondents required an estimated (mean) of 4 ± 11 human eyes per month, and 31 ± 111 eyes per year, although this may change if researchers had ready and routine access to OTR.

### Examining OTR Trends

Although OTR demand in Australia may not be documented or tracked, there is an increasing volume of medical research being conducted,^5^ and government policy and research design is increasingly focused on encouraging translational medicine, with concomitant translation of basic science for human medical use. Such translational work often requires greater quantities of human tissue.

For example, the emergence of increasingly sophisticated molecular and cellular methods has created new demands for OTR. These include multiple methods in biological disciplines, including genomics, epigenetics, and proteomics, as well as immunostaining, cell culture, cell therapy, tissue engineering, and genetic engineering. Similarly, the development of new genetic engineering approaches and single-cell RNA sequencing has created a greater requirement for access to fresh tissue to assess expression profiles of individual or cell-specific profiles.^6^ These innovations enable “big data” approaches, with concomitant ability to undertake analysis of very large datasets to discover statistically significant associations through bioinformatic approaches, for example, Kuiper et al.^7^ and Lin et al.^8^ To fully make use of these scenarios, researchers will be reliant on having access to donor tissue from hundreds or even thousands of individuals. Increased access to OTR might prove a catalyst to such advancements in the biomedical space.

In addition to investigations to further medical knowledge, some of these techniques are being used in clinical trials. The facilities, networks, and the skills of AUEBs position them to not only provide tissue for preclinical experiments and clinical trials but to contribute through assessment of emerging genetic, cellular, and tissue engineered therapies, and drug response changes in tissue or structural changes (e.g. corneal cross-linking). Alongside delivering these therapies to clinicians, some of these therapies will continue to require ocular tissue, provided by AUEBs/Biobanks, for their process and preparation.^9^

Aside from the potential for new techniques and big data, researchers may have specific requirements, some of which will be different to the requirements for donation or locally available OTR. Some experimental techniques require fresh tissue to avoid artifacts resulting from postmortem processes,^6^ for example Lukowski et al.^6^ required retinas to be fewer than 15 hours postmortem. Other techniques require preservation methods not used within AUEB, such as cryopreservation for some immunofluorescence techniques.

## Barriers to OTR

Numerous barriers prevent a steady and robust supply of adequate OTR. We attempt to explore several of these components in this section.

### Diverting Transplant-Surplus to Research

The Price Waterhouse Cooper Report (PWCR)^10^ comments that Australia is meeting surgical ocular tissue need and is in a surplus state. They suggested that Australia should consider exporting surplus tissue that is not required for transplant in Australia. Although they do not explain how they define meeting need, within such a scenario, we propose that a “surplus status” occurs when donor coordinators and/or AUEBs decline eligible donors because scheduled surgical transplant need has been met at that time. As ocular tissue is time sensitive, the AUEBs may not be able to transplant the tissue before it expires. Currently, transplantation is the mainstream use of a donation. Wherever possible, rather than recovering excess tissue, wasting it, and incurring the associated recovery costs (e.g., staffing), the AUEBs respectfully declines the donation.

Although the PWCR intended to focus on Australia's transplant need and services, with their export recommendation as a side emerging theme and a logical first solution that would provide transplant assistance to waiting recipients of another nation, there remains no examination of domestic OTR use as a surplus management strategy. As an alternative or simultaneous proposal, surplus transplant eligible (and ineligible) ocular tissue could still be utilized in Australia, if cost-recovery mechanisms were there to support research. Although not reviewed in the PWCR, OTR need would meet other objectives of the PWCR, such as investment into Australian Research and Development (R&D) and preparing Australia, and the donation sector, for the proposed transition toward tissue engineering and cell therapy. Ironically, such future treatment options are impossible without R&D conducted using human ocular tissue, so retention of some fresh tissue, surplus-to-domestic-transplant need (and recovery of nontransplant grade tissue) could provide greater long-term potential for Australia. Without adequate access to OTR, it is likely that Australian researchers may either go without, being unable to complete their research; modify their experiments to meet access needs; or migrate their work, and the development potential, outside of Australia.

### Domestic and International Obtainment of OTR

Alternatively, researchers in Australia could import OTR from other countries, such as the United States, who do have surplus tissue, that is suitable and consented for research needs.^11^ As the United States also has a robust transplant-exportation system,^12^ such exportation for research may indeed be viable. This would quickly increase access for laboratory-level experimentation. There are no hard laws preventing Australian researchers doing so, assuming adherence to customs and import laws and ensuring that any human trials using imported or research tissue meet the criteria of the Australian regulators.

Although importation appears a viable option, Australian vision science researchers do not appear to import OTR. This may be because of logistics, handling costs, and because the researcher may need to navigate and then manage the relationship with the importer or third-party distributor. The practicalities of transporting tissues from distant exporting nations in the northern hemisphere to Australia is also a challenge, reducing access to the much-needed fresh tissue, resulting in importation of preserved (e.g., hypothermic stored) corneal tissue only. Researchers who import would therefore be restricted to research not requiring fresh tissue (though remaining within an adequate death-to-preservation period).^13^

Within Australia, access to fresh tissue for research may be an issue because of the geographic size of the country. However as most Australian eye researchers are in east coast cities that house most of the AUEBs/Biobanks and recovery allocation practices are based on serving local first, then this access to fresh tissue may not be an issue for most researchers. Perth is the only exception, owing to its isolation on the west coast, with flight times from east to west taking 4.5 hours (on top of recovery, processing, and land transport logistics). Therefore fresh OTR needs, in Perth, are reliant on local self-sufficient services from the Eye Bank of Western Australia. For preserved tissue, Australia has similar death-to-preservation time processes as other countries, and shares similar recovery time, freight cost, and overland transfer times as other larger geographic nations, such as the United States, and as such, domestic preservation methods can be tailored to meet the researchers needs.

Regardless of the nation or the donation recovery systems in place, fresh tissue poses unique logistical and practical challenges, particularly for research protocols requiring fresh tissue within tight timeframes (e.g., within 2 hours of death). Such short time periods are unpractical and unlikely to change, as donation services must allow respectful grieving time to donor families, complete all donation consent steps, and schedule in the AUEB recovery team, prior to the recovery and delivery to the researcher.

Proposing to import OTR from the northern hemisphere, whereas the PWCR simultaneously proposes to export surplus Australian transplant-grade tissue to other countries for transplantation, may sound absurd and inefficient but is not necessarily so. This scenario draws on elements not discussed in the PWCR, whereby the grade of the tissue may predict its use and destination. This, however, raises further questions, such as:

• What is research-grade and transplant-grade tissue?
• Do researchers need access to transplant-grade tissue, as well as nontransplant grade tissue, to conduct comparative studies?
• Should transplant-grade tissue be allocated to recipients today, or go toward research and future therapies for future generations?
• Could donors, who are not triaged through the eligibility process (as they do not meet transplant criteria), also be recovered for research?
• Does death-to-preservation time influence use for transplantation or research?

The current process implies that Australian researchers typically receive nondiseased relatively healthy tissue with some imperfection or donor ineligibility that rendered the tissue not suitable for donation.

### Nontransplant Grade Tissue

As some researchers may specifically seek a diseased eye or tissue from a donor of a specific characteristic (e.g., keratoconus, that would not normally progress to becoming a donor for transplant), the recovery from nontransplant ineligible donors is essential. Access to such donors, with specific criteria, can be problematic, especially in the instance of a rare disease.^14^ The system is currently dependent on the relationships between the AUEB and hospital donor coordinators to liaise and identify possible consenting donors who are ineligible for transplantation who may wish to donate for research specific requests. Unfortunately, identification and willingness to donate do not automatically mean the donation is recovered.^2^ This will depend on funding to the AUEB, which is restricted due to staffing constraints or processing restrictions or space. Additionally, without funding from the researcher or another avenue, the AUEB's willingness to recover tissue and prepare the tissue, at no cost to the researcher, would become a limiting factor for the recovery of OTR.

Development of systems to allow for ineligible transplant donors to donate is important for research,^14^ and may be enhanced through the use of consortia tissue management, assuming multi-AUEB/Biobank-ethical conduct was established. Consortia tissue management occurs when banks of two different natures collaborate to share skills and knowledge to recover from consenting donors of a particular characteristic. For example, the AUEB may be enlisted by the brain bank to recover ocular tissue on behalf of the brain bank. This may occur if researchers are examining the pathway or investigating shared pathology between the brain and the eye, such as in Alzheimer disease, or between the eye and other tissue, such as ocular melanoma and melanoma of the skin, or systematic disease within the same consented donor.

In some instances, AUEBs are contacted by researchers regarding known donors who premortem arrange for their body to be donated to another research bank (e.g., the brain bank), or to a specific research project they participated in during their lifetime. For example, someone who participated in a research program examining macular degeneration may consent for their eye to be examined by the same vision science research team, postmortem, as the final component of their participation in that research project. In these instances, the AUEB staff are contacted to perform the recovery and preparation because they have the appropriate recovery skills. The recovered tissue is prepared and transferred to the predetermined researcher/bank. Such cross-bank consortia, although in occurrence in Australia, are not commonplace within the vision science sector.

### Access to OTR Type

Without a national registry for OTR need and utility, we cannot determine the types of OTR that are routinely required by researchers, for example, the cornea, lens, retina, and so on. Although the Stamer et al.^4^ survey did not ask respondents to clarify the type of tissue they required, they did collect information on the characteristics and section affiliations of their respondents. This may assist in indicating OTR type need of the participating ARVO Members, with their main respondents being cornea (21%), glaucoma (17%), retina cell biology (16%), and retina (10%).

Although Stamer et al.^4^ did not delve further into why the main respondents were from the cornea and glaucoma sectors, we propose that this may be based on their existing relationship with their local eye bank, whose transplant services provide for cornea and glaucoma surgical management. This means that there is a greater likelihood that tissue recovered for those transplants may become OTR if found ineligible for transplant. This is predominantly so if the AUEB tends to recover the corneoscleral rim as routine, as opposed to the whole globe. Although this recovery process works for the transplant sector and prevents additional processing time, cost, and discard of other ocular parts, it does not necessarily work for noncorneal or glaucoma OTR needs. Although we were unable to find evidence, we propose that this may lead to faster and greater R&D success in the cornea and glaucoma sectors, as opposed to other areas of ocular enquiry in years to come.

### Other Access Avenues

Occasionally, consenting nondeceased OTR is recovered from the operating theatre (e.g., the nontransplanted corneoscleral rim). Although the researcher may be known to the AUEB and ethics approved, the recovery is often arranged between the researcher and surgeon for a specific project. Collection practices are outside of the management of the AUEB, with responsibility placed on the transplant facility (hospital, day surgery) and ethics-approved researcher, to ensure donor consent is in place, and safe handling practices are followed prior to handing the OTR to the researcher.^15^ Of note, consenting living recipient/patients may also donate excised OTR (e.g., specimens, tissue of unknown etiology, or corneal buttons). Although such living donation programs and specimen collections are beyond the scope of this article, they indicate another source of OTR.

### Competing Demands for Nontransplant Tissue

The increased interest in lamella surgical techniques in recent decades has also impacted OTR access, with excess recovered corneal tissue being fully utilized by physicians as they perfect these new techniques. Although we were unable to find any published data pertaining to tissue allocation for training use in Australia,^16^ the Eye Bank Association of America (EBAA) states that the United States, “experience[d] a slight decline in the number of corneas provided for research from the highs of 2011 and 2012, [and can be] explained by the concurrent increase in tissue allocated to education and training purposes.” Corcoran^16^ recalls “that this period coincides with the development of more technically challenging endothelial keratoplasty procedures.”

As new surgical techniques continue to be explored, with subsequent surgeon training essential, and animal tissue remaining the only simulated precut training option, then we consider the competing demands with tissue-for-training as a permanent tissue utility requirement in Australia (unless virtual reality simulation systems are developed). Therefore other avenues for OTR access are required to ensure tissue-for-training is simultaneously maintained.

### Unlocking the Barriers to Access

Although there may be a desire to support greater access to OTR, there are several aspects of current practice in Australia that require address:

1. There is a lack of information available to prospective donors and the next-of-kin regarding their option to donate to research.^3^
2. There is no central shared record/registry for OTR other than via the local AUEB/Biobank. This means that researchers may not be aware of tissue available elsewhere, and vice versa.
  ○ Without a shared record/registry, the national OTR need, utility, or importation numbers remains unclear.
  ○ As OTR falls outside of the transplant sector's focal area, there appears to be no central body who would be responsible for the collection of the data or management of a researcher registry.
3. Tissue-for-training remains a competing need. Mechanisms to simultaneously support training and OTR are required, although development of virtual reality simulations could be a useful alternative.
4. Researchers themselves do not routinely factor in OTR cost recovery into grant proposals and project budgets. Therefore they are reliant on sporadic access to no or low-cost domestic tissue, and are unable to afford imports or to pay cost recovery to the AUEB.
5. Researchers with specific donor criteria (e.g., short postmortem times or specific characteristics, such as a female smoker over the age of 50, or a male Pacific Islander with keratoconus) restrict their access to OTR. This is because OTR is not made-to-order and cannot be promised.
6. The conversation regarding demand in Australia needs to be expanded to include examination of OTR need. Its absence to date has resulted in exportation as the only surplus management strategy proposed to the Organ and Tissue Authority in the PWCR.
7. Access to fresh tissue remains reliant on proximity to local AUEBs and their collection methods.
8. The role and perception of the AUEB custodian needs to transition beyond that of a transplant tissue provider to one in which they are the custodian of the donation,^17^ servicing a wide range of donor and public and sector needs, be that transplant, research, and/or training (with the potential to lead the development of simulated training options).

The described barriers are not unique to Australia. The United States is the only known nation to address such barriers through a collaborative EBAA and ARVO initiative. Collectively, they have developed a webportal, *EyeFind*, designed to connect US eye bankers with researchers.^4^^,^^11^^,^^16^^,^^18^

Curcio^18^ suggested that those involved in research must also make changes. She highlighted similar strategies outlined in Principle 9 of The Barcelona Principles (Table),^19^ which are a bioethical framework developed by the Global Alliance of Eye Bank Associations in conjunction with the global eye care and corneal communities. Curcio^18^ proposed that researchers must describe their human tissue selection methods in their manuscripts, and that organizations providing the tissue must be named as authors or acknowledged partners. This highlights the collaborative partnership and ensures that there is significant demonstration of criteria for ethical and appropriate recovery and allocation.

**Table.** The Barcelona Principles 2018^19^

PRINCIPLE 9: Ensure Ethical Practice and Governance of Research (Nontherapeutic) Requiring Cells Tissue and Organs (CTO). Strategy:
I. Ensure consent for research. II. Provide tissue to research and technical development projects where all parties demonstrate ethically sound practices and processes. III. Ensure any intended research for which CTO is requested has been designed, and will be conducted, in accordance with jurisdictional law, and regulations that govern the ethical use of human tissue (inclusive of the Declaration of Helsinki/International Ethical Guidelines for Health-related Research Involving Humans), and: a. obtain approval from a qualified human research ethics committee. b. work with scientific journals and peer associations/societies to promulgate scientific standards that honor the ethical consent of CTO for research. IV. Researchers should verify that the eye bank providing the tissue has appropriate credentials, policies, and practices, and is transparent and open to scrutiny (e.g., demonstrating their ethical consent process for obtaining and allocating CTO for research or further attenuation/commercialization). V. Scientific journals should establish a mechanism to confirm research is conducted on ethically obtained CTO.

## Reform Strategies

The research, transplant, and donation sectors are interconnected, unable to improve, progress, or resolve issues without co-operation. Researchers are reliant on OTR to develop new treatment options. The transplant sector is simultaneously reliant on R&D to provide new treatment options. Finally, the donation sector is also reliant on the discovery of new technologies to reduce the long-term burden and strain on donation services. This will ultimately lead to a point in which there is a reduced need for donations, as other approaches can be used.

Regulation has not kept pace in the ever-changing research and deceased donation sectors,^17^ and is unlikely to do so for some time. Therefore we propose the sector itself take a leadership role and promote change by engagement of researchers, organ and tissue professionals, and transplant professionals to develop a policy and practice reform strategy that improves access to OTR for Australian researchers, while simultaneously ensuring such access does not compromise donor wishes, hinder transplant needs, or undermine tissue for training needs or potential exportation. We believe, if done in a systematic manner, Australia could become a leader in OTR practice and retain a viable vision science research sector.

We propose a series of policy and practice strategies are developed to unblock the barriers we described earlier via stakeholders, including the Organ and Tissue Authority, AUEBs/Biobanks, vision science researchers, bioethicists and biological health lawyers, and the professional peer associations of these groups. These strategies could include:

1. A recommendation that all researchers automatically factor in average OTR costs into their grants and budgets or researchers work with their funding bodies to develop new OTR funding avenues.
2. Adherence to The Barcelona Principles: Principle 9 (Table)^19^; all published research to include information regarding where and how they obtained OTR and its ethical origin.
3. Placement of an additional “Register for Research” box on the national Medicare donor register page. This will assist in identifying OTR donors and could assist in identifying donors with specific disease characteristics.^14^
4. Development of a public information campaign, reemphasizing consent for research options to donate nontransplant grade tissue to research, and/or research-only donations. Information could be made available alongside information on donation for transplantation.
5. Provision of information via a health provider (e.g., a general practitioner, ophthalmologist, optometrist, nurse) directly to patients, outlining their option to donate for research. This could also assist in increasing rare, diseased, or specific demographic donation requests,^20^^,^^21^ as otherwise such donors may not consider donation on their death.
6. Allowance of those ineligible for transplantation, who would otherwise exit the donation pathway preconsent, to be consented and recovered as OTR donation only.
  a. Funding options will need to be addressed regarding provision of OTR at either a no cost and/or at a fee subsidized by the Australian Government or another avenue, as such methods may risk the sustainability of the AUEB/Biobank. Our proposal is outlined in Figure 2.
7. Development of a national co-operatively managed register to allow ethics-approved researchers to connect directly with their AUEB/Biobank to match their OTR need with available donations. This system could allow:
  a. The AUEB/Biobank to arrange for ethics-approved imports or cross-state/territory OTR, as required by researchers. This would alleviate navigation issues for the researcher, who would otherwise have to register with multiple providers, work with distributors, manage the logistics and confirmation of the ethical validity of the donation and involved parties. This ensures Australian research, involving domestic or foreign donors, meet the ethical norms and legal principles that govern Australia, and those outlined within The Barcelona Principles, without limiting the potential of Australian R&D.
  b. Ensure Australia has an OTR tracking and data monitoring system in place.
  c. Improve relations between AUEB/Biobank and researchers.
  d. Open up opportunities for collaboration with other national and international eye banks, researchers, consortia, and donation agencies.
  e. Ensuing cross-sector transparency to the Australian public and government.

## Conclusions

Although access to human tissue of any quantity and size is an end-of-life donation, rather than a consumable that can be demanded, if there is an unmet need for OTR, and there are donors whose donation wishes have not been met, then steps could be taken to ethically and practically meet the wishes and needs of all parties.

Barriers to OTR include research budgeting and other cost avenues, limited access to the tissue—and in particular fresh tissue—lack of donor information,^3^ and a system that has historically focused on transplantation. Although regulatory change is a primary requirement, it is unlikely to occur in the short-term. As such, we call on sector stakeholders to strengthen their relations as leaders of this field and work collaboratively to resolve and remove nonregulatory barriers to OTR. In doing so, Australia will retain and build its position as a prominent leader in the vision sciences.

## References

[1] Australian Government. *2016 National Research Infrastructure Roadmap.* Available at: https://docs.education.gov.au/system/files/doc/other/ed16-0269_national_research_infrastructure_roadmap_report_internals_acc.pdf. Accessed June 4, 2019.

[2] Machin H, Pollock GA Eye banking. In: Marsden J, ed. *Ophthalmic Care*. 2nd ed London: M&K Publishing; 2016: 289–312.

[3] Curcio CA Declining availability of human eye tissues for research. *Invest Ophthalmol Vis Sci*. 2006; 47: 2747–2749. 16799009 10.1167/iovs.05-0978

[4] Stamer WD, Williams AM, Sflugfelder S, Coupland SE Accessibility to and quality of human tissue for research: a cross-sectional survey of ARVO Members. *Invest Ophthalmol Vis Sci*. 2018; 59: 4783–4792. 30304462 10.1167/iovs.18-25319

[5] Ware M, Mabe M The STM Report. An overview of scientific and scholarly journal publishing. 4th ed The Netherlands: International Association of Scientific, Technical and Medical Publishers; 2015: 180.

[6] Lukowski S, Lo C, Sharov A, et al. Generation of human neural retina transcriptome atlas by single cell RNA sequencing. Cold Spring Harbor, NY: Cold Spring Harbor Laboratory;2018:bioRxiv 425223.

[7] Kuiper JJW, Setten JV, Devall M, et al. Functionally distinct ERAP1 and ERAP2 are a hallmark of HLA-A29-(Birdshot) Uveitis. *Hum Mol Genet*. 2018; 27: 4333–4343. 30215709 10.1093/hmg/ddy319 PMC6276832

[8] Lin H, Long E, Ding X, et al. Prediction of myopia development among Chinese school-aged children using refraction data from electronic medical records: a retrospective, multicentre machine learning study. *PLoS Med*. 2018; 15: e1002674. 30399150 10.1371/journal.pmed.1002674 PMC6219762

[9] Porzionato A, Stocco E, Barbon S, Grandi F, Macchi V, De Caro R Tissue-engineered grafts from human decellularized extracellular matrices: a systematic review and future perspectives. *Int J Mol Sci*. 2018; 19: E4117. 30567407 10.3390/ijms19124117 PMC6321114

[10] Organ and Tissue Authority. Final report: analysis of the Australian eye and tissue sector. 2016 Section 3.1.3. 15–17. https://donatelife.gov.au/about-us/corporate-information/government-reports . Accessed April 8, 2020.

[11] Eye Bank Association of America. 2019 Eyefind. Available at: https://www.arvo.org/journals-and-publications/EyeFind/. Accessed May 2, 2019.

[12] Gain P, Jullienne R, He Z, et al. Global survey of corneal transplantation and eye banking. *JAMA Ophthalmol*. 2016; 134: 167–173. 26633035 10.1001/jamaophthalmol.2015.4776

[13] Williams AM, Allingham RR, Beckwith HS, Liu PJ, Santiago-Turla C, Muir KW Patient and family attitudes about an eye donation registry for research. *Curr Eye Res*. 2013; 38: 945–951. 23767667 10.3109/02713683.2013.800890

[14] VonDran M, Thomas JA, Freund MP, et al. The national disease research interchange and collaborators on: what are the major hurdles to the recovery of human tissue to advance research? *Biopreserv Biobank*. [ahead of print] 2016 Available at: 10.1089/bio.2016.29014.mvd. PMC5690877 27845557

[15] Eye Bank Association of Australia and New Zealand. 2016 National guidelines: a resource for Australian hospitals, operating theatres and day surgery staff regarding the care and handling of human tissue for ocular transplantation. Available at: http://www.ebaanz.org/wp-content/uploads/2016/03/EBAANZ-NATIONAL-GUIDELINES-2016-For-the-care-and-handling-of-HTO-FINAL.pdf . Accessed May 2, 2019.

[16] Corcoran KP Accessibility to and quality of human eye tissue for research: a cross-sectorial survey of ARVO members–EBAA commentary. *Invest Ophthalmol Vis Sci*. 2018; 59: 4793–4795. 30304460 10.1167/iovs.18-25631

[17] Aparicio L, Lipworth W, Then SN, et al. Biobanking of blood and bone marrow: emerging challenges for custodians of public resources. *J Law Med*. 2013; 21: 343–350. 24597383

[18] Curcio CA A new online portal will match eye banks with researchers seeking human ocular tissues. *Invest Ophthalmol Vis Sci*. 2018; 59: 4796–4797. 30304461 10.1167/iovs.18-25632 PMC6166890

[19] The Barcelona Principles: an agreement on the use of human donated tissue for ocular transplantation, research, and future technologies. *Cornea*. 2018; 37: 1213–1217. 30198942 10.1097/ICO.0000000000001675

[20] William AM, Allingham RR, Stamer WD, Muir KW Eye care professionals’ perspectives on eye donation and an eye donation registry for research: a single-institution, cross-sectional study. *Curr Eye Res*. 2016; 41: 867–871. 26287578 10.3109/02713683.2015.1056376

[21] Williams AM, Stamer WD, Allingham RR Increasing the availability and quality of donor eyes for research. *JAMA Ophthalmol*. 2016; 134: 351–352. 26768017 10.1001/jamaophthalmol.2015.5492 PMC4932832

